# The Effect of Different Dietary Sugars on the Development and Fecundity of *Harmonia axyridis*

**DOI:** 10.3389/fphys.2020.574851

**Published:** 2020-09-15

**Authors:** Yan Li, Shasha Wang, Yongkang Liu, Yuting Lu, Min Zhou, Su Wang, Shigui Wang

**Affiliations:** ^1^College of Life and Environmental Sciences, Hangzhou Normal University, Hangzhou, China; ^2^Institute of Plant and Environment Protection, Beijing Academy of Agricultural and Forestry Sciences, Beijing, China

**Keywords:** *Harmonia axyridis*, artificial diets, glucose, trehalose, fecundity

## Abstract

The aim of this study was to screen synergistic substances included in existing artificial feeds in order to improve the fertility and survival rate of *Harmonia axyridis* (Pallas) (Coleoptera: Coccinellidae), an efficient pest predator. To this end, we analyzed the potential effects of glucose and trehalose on the growth, development, and reproduction of *H. axyridis* and evaluated the effect of three different artificial feeds on the energy stress of *H. axyridi*s. The artificial diets contained fresh pork liver, honey, sucrose, vitamin C, and royal jelly, which was marked it as Diet1. The glucose was added to diet1, which was marked it as diet2, while adding trehalose to diet1 was marked as diet3. The pre-oviposition period of *H. axyridi*s on Diet 1 was slower than that of Diet 2 and Diet 3. Additionally, the spawning quantity and incubation rate of insects on Diet 2 and Diet 3 were significantly higher than that of those on Diet 1. Finally, the larval developmental time on Diet 1 was significantly slower than that of Diet 2 and Diet 3. These results indicate that the addition of an appropriate amount of glucose or trehalose may affect positively the growth, development, and reproduction of *H. axyridis*. In addition, further studies showed that ATP, amino acids and fatty acids content in the *H. axyridis* also increased after the addition of the synergistic substance. All these results show that proper adjustment of stored energy anabolic and catabolism is important to maintain the metabolic balance of the insect’s entire life cycle and the addition of glucose or trehalose has a certain effect on the life indicators of *H. axyridis.*

## Introduction

Agricultural and forestry crop pests seriously threaten the quality and yield of agricultural production, causing huge economic losses (Hansen et al., 1999). Although chemical control is used for the direct elimination or extermination of agricultural and forestry crop pests, the use of pesticides on a large scale causes severe environmental pollution, endangers human health (Youn et al., 2003; Katsarou et al., 2005; Garratt and Kennedy, 2006), and results in the resistance of many pests (Puinean et al., 2010; Kavi et al., 2014; Bass et al., 2015; Saddiq et al., 2015). It is well-known that natural enemy insects are important biological agents in global agricultural ecosystems (Juen et al., 2012; Lu et al., 2012). However, they often face seasonal natural food shortages. In addition, the manual feeding of natural food requires the maintenance of a three-level nutrition chain, which increases both the feeding and the biological control cost (Liu et al., 2013). An artificial diet is the key to avoid the seasonal restrictions of natural food and meet the needs of various experiments. Agricultural pests, such as Hemiptera, Coleoptera, Lepidoptera, and a variety of economic insects, such as the *Asian corn borer* (Fadamiro et al., 2005) are some of the insect species fed artificial diets.

*Harmonia axyridis* (Pallas) (Coleoptera: Coccinellidae) is an efficient pest predator (Koch, 2003). A large number of studies have shown that *H. axyridis* is an important insect in the integrated pest management strategy (Brown et al., 2011; Castro et al., 2011; Luo et al., 2014). As early as 1916, *H. axyridis* was released as a biological control insect in orchards, farms, and greenhouses (Michaud, 2002; Majerus et al., 2006; Brown et al., 2011; Van, 2012), but so far there are still natural prey that cannot sustain the ladybugs throughout the year (Zhang et al., 2014). It is difficult to maintain a sufficient *H. axyridis* supply in the biological control of insects, especially when it comes to large-scale breeding populations in laboratories (Zazycki et al., 2015). In large-scale insect propagation, feeding aphid diets in captivity is an expensive and time-consuming method (Bonte et al., 2010). Therefore, it is necessary to develop artificial diets that *H. axyridis* can be supplemented with, in order to achieve the low-cost and continuous expansion of this insect that will facilitate the effective control of pests (Cheng et al., 2018). In response to this problem, various artificial insect diets have been exploited and proposed (Cohen, 2001; Castane and Zapata, 2005). Research on artificial diets for *H. axyridis* has ranged from exploring original insect source components such as *Tenebrio molitor* and *Trichogramma dendrolimi* Matsumura (Guo and Wan, 2001) to non-insect sources with fresh pig liver as the main component. Many studies have proposed that although insects can be bred with artificial feedstuffs with some success, in many cases they lose their ability to adapt and reproduce, which has led to a delayed development and lower fertility (Denno and Fagan, 2003; Wilder et al., 2011; Schmidt et al., 2012). The previous research, suggested that the main reason for the decline in the hatchability and reproductive capacity of *Spodoptera exigua* was long-term indoor mating, in subsequent research, these decreases were attributed to irrational feed nutrition (Li et al., 2002).

Studies on *Microplitis mediator* in bee cocoons have revealed that feeding glucose can enhance the reproductive efficiency and potential of the bees. It has been suggested that the main reason for this is that a carbohydrate food supplement provides mature eggs with the energy they need as they do not need to resort to their lipid reserves to acquire energy; additionally, the accumulation of lipids in the female’s body is directly used in oogenesis and egg maturation (Huang et al., 2015). Glucose is the most widely used sugar by insects for energy production and the supply of large molecular precursors and is a signaling molecule in the liver and adipose tissue (Vaulont et al., 2000). As a direct energy substance, glucose can be used better by females for egg maturation (Huang et al., 2015). Some reports showed that glucose metabolism and its regulation are particularly important for termite reproduction; glucose is particularly important for feeding adult females as it provides them with energy for reproduction and promotes egg maturation (Thompson, 2003). The energy metabolism of insects, which is otherwise similar to that of other animals, has a unique feature; the synthesis and utilization of trehalose (Tang et al., 2010). The regulation of trehalose metabolism and the control of glucose utilization are important in terms of energy (Tang et al., 2010). Trehalose can effectively prevent the denaturation and functionality loss of proteins under adverse conditions (Haque et al., 2015). In addition, studies have shown that the trehalose content affects food choices and feeding behavior. The physiological role of trehalose as insects’ blood sugar during their reproductive processes has been reported previously (Lu et al., 2019). A parallel relationship between the hemolymph trehalose levels and ovarian maturation was observed, suggesting that trehalose supplies the energy required for the reproductive cycle processes (Huang and Lee, 2011). Trehalose is a readily accessible energy source that can be used as required (Shi et al., 2017).

In addition, studies have shown that the main parts of insects that produce and store glycogen and trehalose are fat bodies, ovaries, and flying muscles (Tang et al., 2012). Trehalose is the energy fuel for vitellogenin (Vg) formation and oocyte maturation (Lu et al., 2019). Therefore, it is speculated that eating artificial feedstuffs with glucose or trehalose could provide insects with sufficient energy to act on the ovary, thereby promoting the development of their ovaries and increasing spawning. The population proliferation of insects is based on individual reproduction, and reproductive success depends on the Vg synthesis of the fat body and on oocyte development (Tufail and Takeda, 2008; Tufail et al., 2010). Vg is the main storage protein precursor of the ova of many animals, the energy reserve of many ovipara (Zhang et al., 2017), and plays an important role in the reproduction of oviparous vertebrates and invertebrates (Liu et al., 2013).

The aim of this study was to supplement insect diets with potentially synergistic substances while meeting their minimum nutritional requirements. Additionally, we aimed to discover and screen more effective and high-quality artificial feedstuffs for natural enemy insects that would lead to higher proliferation, thereby providing technical support for their release. Based on previous reference on artificial feedstuffs (Yang et al., 2013), we used glucose and trehalose as energy supplying nutrients for insects. By measuring the growth, development, reproductive ability, and substance content indicators in the bodies of *H. axyridis*, we evaluated the potential role of glucose and trehalose on their growth, development, and reproduction.

## Materials and Methods

### Insects

*Harmonia axyridis* individuals were raised in the Key Laboratory of Animal Adaptation and Evolution of Hangzhou Normal University. All insects were bred in a growth chamber maintained at 23 ± 2°C, with 68 ± 5% relative humidity (RH), and a photoperiod of 16:8 h (L: D). *H. axyridis* individuals were fed *Aphis medicaginis* at a fixed time every day. After oviposition, the eggs were collected 2–3 times daily and placed in a 7 cm Petri dish containing filter paper; distilled water was sprayed onto the filter paper once a day to preserve its humidity. After the eggs hatched, we used a writing brush to gently move the hatched larvae into individual larva rearing boxes containing aphids or artificial feedstuffs (4 cm × 3 cm × 2 cm). It was necessary to keep them alone in order to prevent cannibalism and competition among individuals. After being reared to emergence, the adults (12 females and 8 males in each box) were placed in adult feeding boxes containing aphids or artificial diets. The diet in all feeding boxes was changed once a day and wet cotton balls were replenished.

### Preparation of Artificial Diets

In this experiment, we used the artificial feed formula prepared by Yang et al. (2013) as reference material; we adapted it by optimizing the addition of ingredients and improving their ratio (Yang et al., 2013). Three artificial feed formulae were designed, namely Diet 1, Diet 2 and Diet 3, CK represents *Aphis medicaginis* used as the control. The raw materials were weighed and mixed according to the number of insects in each group, in order to prepare a semi-fluid feed and were stored in a refrigerator at −20°C. For a detailed component analysis of each formula, see Table 1.

**TABLE 1:** Specific ingredients of the artificial feed formula of the *H. axyridis*.

**Ingridients**	**Diet 1**	**Diet 2**	**Diet 3**
**Fresh pork liver**	64%	64%	64%
**Honey**	13.5%	13.5%	13.5%
**Vitamin C**	3.5%	3.5%	3.5%
**Sugar**	13.5%	13.5%	13.5%
**Royal jelly**	5.5%	5.5%	5.5%
**Glucose**	–	12%	–
**Trehalose**	–	–	20%

### Experimental Method

#### Determination of the Tissue and Hemolymph Sugar Contents of *H. axyridis* Larvae

At least 50 early fourth instar larvae were collected from each of the three groups, which fed with Diet 1, Diet 2, and Diet 3. After having fed for 12, 24, and 48 h, the larvae were placed on ice. After the larval ability decreased, their feet were punctured by a medical anatomical needle. Hemolymph was collected with a capillary tube and transferred to an Eppendorf (EP) tube (Eppendorf, Hamburg, Germany) containing anticoagulant (Wang et al., 2007). Each tube contained the hemolymph of seven larvae. After hemolymph extraction, the tissue of larvae was placed into a new EP tube; each tube contained the tissue of three larvae. Each of the experiments was performed in three biological replicates and three technical replicates.

The above samples were ground with phosphate buffered saline (PBS) and sonicated. Subsequently, 350 μL of the supernatant was collected and ultracentrifuged at 20,800 *g* for 60 min at 4°C. The remaining supernatant was used to determine the protein, trehalose, and glycogen contents. The trehalose content was measured using the anthrone method. Briefly, we took 30 μL of the centrifuged supernatant sample, added 30 μL of 1% H_2_SO_4_, placed it in a water bath at 90°C for 10 min, and then in an ice bath for 3 min. After adding 30 μL of 30% KOH, the sample was incubated again at 90°C in a water bath for 10 min and in an ice bath for 3 min. Then, 600 μL of developer was added and the sample was placed in a water bath at 90°C for 10 min and then cooled in an ice bath. The absorbance of the sample was measured at 630 nm using a microplate reader. A glucose assay kit (Sigma-Aldrich, St. Louis, MO, United States) was used to measure the glucose content by taking 150 μL of 20,800 *g* centrifuged supernatant and 20,800 *g* of pellet suspension, according to the manufacturer’s instructions. After incubation in water bath at 37°C for 30 min, 300 μL of 2N H_2_SO_4_ was added to stop the reaction, and the absorbance of the samples was measured at 540 nm using a microplate reader. For the determination of the glycogen contents, 160 μL of supernatant obtained after centrifugation at 1,000 *g* was added to 600 μL of anthrone sulfate reagent, and the mixture was incubated at 90°C for 10 min and then cooled in an ice bath. The absorbance of the sample was measured at 625 nm using a microplate reader. The protein content was determined using the BCA Protein Assay Kit (Beyotime, Shanghai, China).

#### Observation of the Developmental Duration and the Body Weight Index of *H. axyridis* Larvae

At least 100 eggs were collected from each group. We measured the developmental period from the first instar to the pupal stage and monitored the development of *H. axyridis* every day. At the same time, the body weight of the larvae that fed on different artificial feeds was recorded. Electronic scales (METTLER TOLEDO, Shanghai, China) were used to weigh the first, second, third, and fourth instar larvae. As the larvae were small, 10 larvae that fed on the same feed and were at the same developmental stage were weighed each time. Then, we recorded the average weight of the three groups; the above experiment was repeated three times.

#### Observations on the Pre-oviposition, Oviposition Rate, and Fecundity of *H. axyridis*

We collected at least 50 pairs of *H. axyridis* adults from each group. Each group was provided with five feeding boxes for 42 days. The time from the first day of the emergence of *H. axyridis* to the oviposition time was considered as the pre-oviposition. Then, the daily oviposition of adults that had fed on different diets and aphids was counted and recorded. We recorded the average of three breeding rounds as the final spawning result. From each round, we selected approximately 100 eggs and repeated this process three times per group. Then, we calculated the hatchability of the eggs by observing and recording the number of first instar larvae after 1 day of incubation.

#### Determination of the Sugar and Substance Contents of *H. axyridis* Adults

We collected at least 50 pairs of the adults from each group at the beginning of the emergence stage. Then, we measured the sugar and substance contents of females that had fed for 48 and 72 h. The sugar content detection method was the same as the one described in Section “Determination of the Tissue and Hemolymph Sugar Contents of *H. axyridis* Larvae.” We used the GLYCERINE kit, the Non-esterified Free fatty acids assay kit (NEFA), and the Total Amino Acid assay kit to measure the glycerine, free fatty acid, and adenosine 5′-triphosphate (ATP) contents. The above kits are collected from Nanjing Jiancheng Bioengineering Institute.

#### Ovarian Development in *H. axyridis* Adults

We collected at least 50 pairs of the adults from each group at the beginning of the emergence stage and fed them Diet 1, Diet 2, and Diet 3. The ovaries of *H. axyridis* that had fed for 3, 5, and 7 days were dissected. The vivisection of female *H. axyridis* was conducted in saline; first, we cut off their wings and head and then we attached the insect bodies to the anatomical box (AGAR dish). Under the Leica EZ4HD stereoscopic microscope (Leica, Wetzlar, Germany), we cut along the middle back of the adults to the end of the abdomen and removed the organs and tissues of non-reproductive systems, such as the digestive tract and the fat body.

#### Determination of the Relative Expression of the VgR and Vgs Genes in *H. axyridis*

We collected at least 50 pairs of the adults from each group at the beginning of the emergence stage. Ha-rp49 was used as an internal reference gene and detected the relative expression of the *VgR*, *Vg1*, and *Vg2* genes in their adults.

First, total RNA was extracted from the adults of *H. axyridis* by a Trizol-based method. Each tube sample was homogenized with 800 μL of TRIzol reagent (Invitrogen, Carlsbad, CA, United States) according to the manufacturer’s instructions. A Thermo Scientific NanoDrop 2000 UV-Vis spectrophotometer (Thermo Fisher Scientific, Inc., Waltham, MA, United States) was used to determine the RNA quality and quantity. Complementary DNA (cDNA) was synthesized from 1 μg of total RNA using the PrimeScript^TM^RT reagent Kit with gDNA Eraser (perfect Real Time) (Takara Bio, Inc., Kusatsu, Japan) according to the manufacturer’s protocol.

The cDNA was diluted fivefold for subsequent quantitative real-time polymerase chain reaction (qRT-PCR) analyses. qRT-PCR was carried out in 20 μL reactions containing 1.0 μL cDNA, 10 μL SYBR Green Premix Ex Taq (Takara Bio, Inc.), 1 μL forward primer (10 μM), 1 μL reverse primer (10 μM), and 7 μL nuclease free water using the Bio-Rad CFX96^TM^ Real-Time PCR Detection System (Bio-Rad Laboratories, Inc., Hercules, CA, United States). Then, we conducted a melting curve analysis (from 60 to 95°C) to ensure the consistency and specificity of the amplified product. All samples were normalized to the threshold cycle value for QHa-rp49 mRNA, which was chosen as an invariant control. Information on the primer sequences of the *H. axyridis* genes is shown in Table 2.

**TABLE 2:** Sequences of qRT-PCR primers for *VgR* and *Vgs* genes.

**Gene**	**Forward (5′–3′)**	**Reverse (5′–3′)**
HaVgR	TGTAGGAGGCGAAGCAATGAT	TGGGATGTGACAGGGAAATAA
HaVg1	GCAACAGAGTCCGTGGTCTTT	GCTGCTTTCACCGTTCTTCAA
HaVg2	CAATCAAAACTCAAGCA	GTCAAAAACTGGATGGAC
	AGGAGA	AACAA
QHarp49	GCGATCGCTATGGAAAACTC	TACGATTTTGCATCAACAGT

### Statistical Analyses

All the data were analyzed using a one-way ANOVA with the statistical software package version 7.0 (StatSoft, Inc., Tulsa, United States). Multiple comparisons of means were conducted using Tukey’s test. Differences between means were deemed to be significant when *P* ≤ 0.05. Statistical analysis was performed with STATISTICA 8.0 and Sigma Plot 10.0. The qRT-PCR data were processed using the 2^–△△CT^ method (Livak and Schmittgen, 2001).

## Results

### Tissue and Hemolymph Sugar Contents of *H. axyridis* Larvae

**FIGURE 1:**
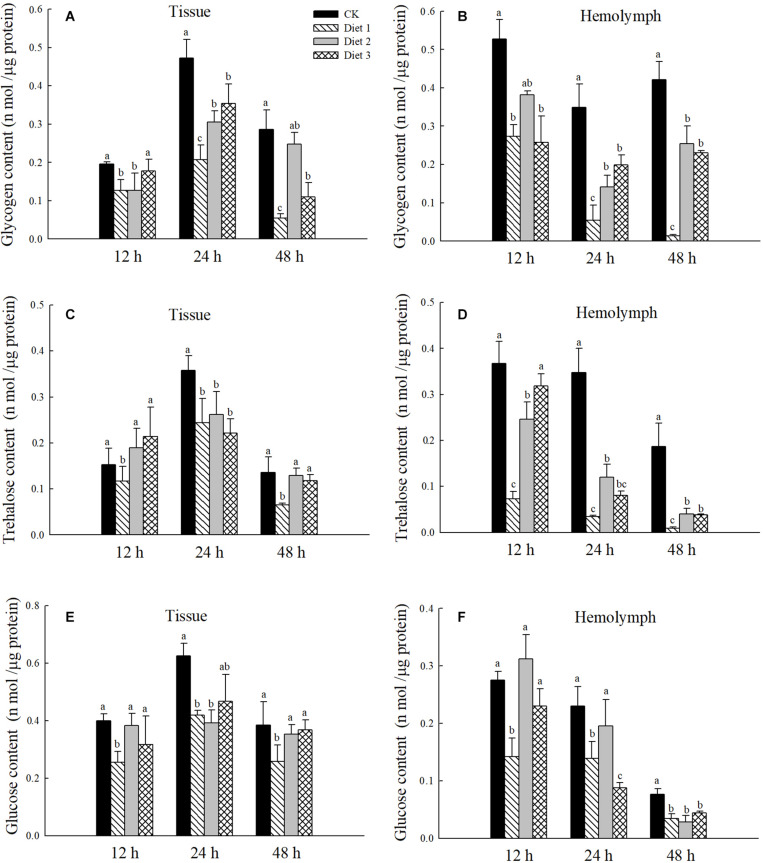
After 12, 24, and 48 h of fed with three kinds of artificial feedstuffs, the sugar content in the larval tissue and hemolymph of *H. axyridis*. **(A)** The glycogen content in the tissue. **(B)** The trehalose content in the tissue. **(C)** The glucose content in the tissue. **(D)** The glycogen content in the hemolymph. **(E)** The trehalose content in the hemolymph. **(F)** The glucose content in the hemolymph. Line bars represent standard error of the mean. Bars with different letters are significantly different (Tukey–Kramer tests, *P* < 0.05). Numbers of samples for each treatment of hemolymph = 7. Sample size for each treatment of tissue = 3.

The tissue and hemolymph glycogen content after 24 and 48 h feeding on the Diet 2 and Diet 3 were significantly higher than that of the Diet 1 (*P* < 0.05) (Figures 1A,D). The trehalose content in the tissues of larvae fed on Diet 1 for 24 and 48 h Diet 1 was significantly lower than that of the Diet 2 and Diet 3 (*P* < 0.05) (Figure 1B). The trehalose content in the hemolymph of the Diet 1 was lower than that of the Diet 2 and Diet 3 (*P* < 0.05) (Figure 1E). The glucose content in the tissues of all groups did not change significantly after having fed for 24 h. However, the glucose level of Diet 1 was significantly lower than those of Diet 2 and Diet 3 (*P* < 0.05) after 48 h (Figure 1C). After 12 and 24 h of feeding, the glucose content in the hemolymph of Diet 1 was significantly lower than that of Diet 2 and Diet 3, while there were no significant differences among the insects of the three groups after they had fed for 48 h (*P* < 0.05) (Figure 1F).

### Developmental Time and Larval Weight

**FIGURE 2:**
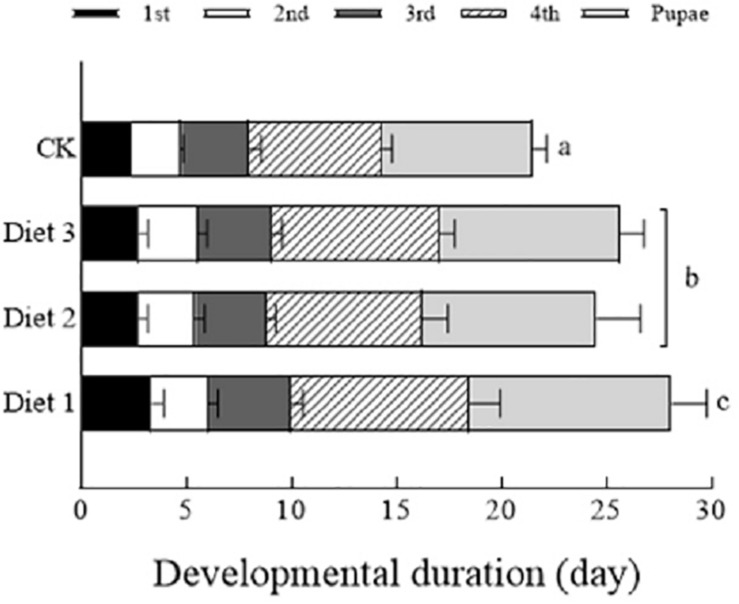
Development periods of larvae to pupae after feeding with different artificial feeds. The “1st” represents the 1st instar larval stage, “2nd” represents the 2nd instar larval stage, “3rd” represents the 3rd instar larval stage, “4th” represents the 4th instar larval stage, and “Pupae” represents the entire pupal stage. Line bars represent standard error of the mean. Bars with different letters are significantly different (Tukey–Kramer tests, *P* < 0.05). Sample size for each treatment = 30.

We observed and recorded the developmental time of the larvae and pupae that had fed on different artificial diets. As is shown in Figure 2, the effect of different diets on the developmental time of the larvae was significant (*P* < 0.05). The developmental times of the larvae fed on of Diet 2 and Diet3 were much shorter than that of Diet 1 (25.8, 24.5, and 28.1 days, respectively). The developmental time of each of the aforementioned groups was longer than that of the group that fed on aphids, which was about 21.5 days. As is presented in Table 3, the *H. axyridis* that fed on the three diets were lighter than those that fed on aphids. Additionally, Diet 2 and Diet 3 individuals weighed more than Diet 1 individuals. More specifically, the differences were significant at the third and fourth instar and at the pupal stage (*P* < 0.05). On weight of with different lowercase letters are significantly different.

**TABLE 3:** Effect of different artificial diets on weight of *Harmonia axyridis* pupae.

**Prescriptions**	**1st (mg)**	**2nd (mg)**	**3rd (mg)**	**4th (mg)**	**Pupae (mg)**
*Aphis medicaginis*	0.68 ± 0.071 a	2.3 ± 0.90 a	10 ± 0.48 a	17.97 ± 0.14 a	23.622 ± 0.84 a
Diet 1	0.63 ± 0.64 a	1.2 ± 0.41 c	4.1 ± 0.42 d	10.74 ± 0.32 c	19.467 ± 0.76 b
Diet 2	0.64 ± 0.020 a	1.6 ± 0.20 b	8.7 ± 0.35 a	15.58 ± 0.24 b	22.833 ± 0.50 a
Diet 3	0.66 ± 0.03a	1.9 ± 0. 37 ab	6.6 ± 0. 24 c	16.03 ± 0. 31 b	21.967 ± 0. 16 a
					

### Pre-oviposition Period, Fecundity, and Hatching Rate Indicators of *H. axyridis* Adults

**FIGURE 3:**
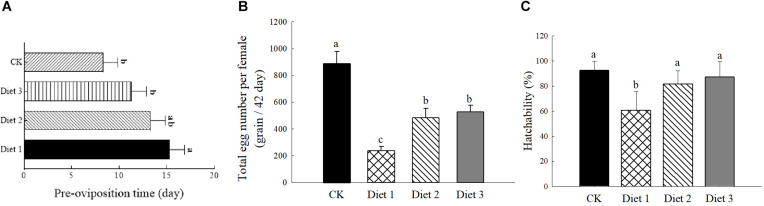
Pre-laying period, egg-laying number and hatching rate of *H. axyridis*. **(A)** Pre-oviposition period, numbers of samples for each treatment = 36. **(B)** In a single trial lasting 42 days of eggs laid by *H. axyridis*, numbers of samples for each treatment = 36. **(C)** Hatching rate, numbers of samples for each treatment = 50. Line bars represent standard error of the mean. Bars with different letters are significantly different (Tukey–Kramer tests, *P* < 0.05).

The results presented in Figure 3 show that the different artificial diets had a significant effect on the pre- oviposition, egg production, and hatching rate of *H. axyridis* (*P* < 0.05). The pre-oviposition period on Diet 1 (15.33 ± 1.52 days) was longer than that of Diet 2 and Diet 3 (13.33 ± 1.17 and 11.75 ± 1.72 days, respectively) (Figure 3A). Additionally, the fecundity (Figure 3B) and the incubation fertility (Figure 3C) of Diet 2 (483.50 ± 70.88 eggs per female, 81.69 ± 10.60%) and Diet 3 (527.66 ± 49.65 eggs per female, 87.00 ± 12.64%) were significantly higher than those of Diet 1 individuals (236.92 ± 32.56 eggs per female, 60.67 ± 15.23%).

### Chemical Contents of Adults

**FIGURE 4:**
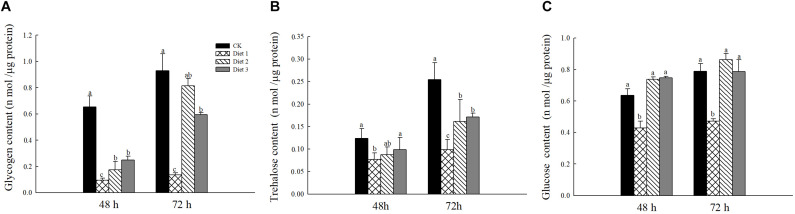
Sugar content in female *H. axyridis* after 48 and 72 h of feeding on artificial feedstuffs. **(A)** represents the glycogen content. **(B)** Represents the trehalose content. **(C)** Represents the glucose content. Line bars represent standard error of the mean. Bars with different letters are significantly different (Tukey–Kramer tests, *P* < 0.05).

The glycogen content on Diet 2 and Diet 3 was significantly higher than that of Diet 1 (*P* < 0.05; Figure 4A). In all time treatments, i.e., 48 and 72 h feeding on different artificial diets, trehalose and glycogen contents of the adults fed on Diet 2 and Diet 3 were significantly higher than that of Diet 1 (*P* < 0.05; Figures 4B,C).

**FIGURE 5:**
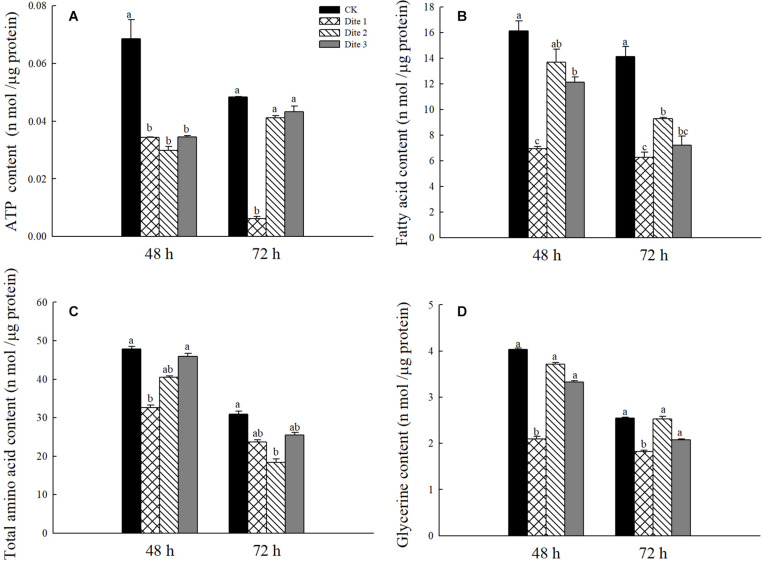
Substance content in female *H. axyridis* after feeding on artificial feedstuffs for 48 and 72 h. **(A)** ATP content. **(B)** Fatty acid content. **(C)** Total amino acid content. **(D)** Glycerin content. Line bars represent standard error of the mean. Bars with different letters are significantly different (Tukey–Kramer tests, *P* < 0.05).

In addition, we tested the content of associated chemical in adult *H. axyridis* after 48 and 72 h of feeding on different artificial diets. As is shown in Figure 5, after 72 h feeding on Diet 2 and Diet3 the ATP content of the adults was significantly higher than that on the adults fed with Diet 1 while there was no significant difference after feeding for 48 h (*P* < 0.05; Figure 5A). After 48 and 72 h feeding on Diet 2 and Diet 3, the fatty acid content was significantly higher than that of Diet 1 (*P* < 0.05; Figure 5B); In this experiment, there was no significant difference among the total amino acid contents of the three groups after they had fed on an artificial diet for 48 and 72 h (*P* < 0.05; Figure 5C). Finally, the glycerol content of Diet 2 and Diet 3 individuals, after they had fed for 48 and 72 h, was significantly higher than that of Diet 1 individuals (*P* < 0.05; Figure 5D).

### Ovarian Development of the Adults

**FIGURE 6:**
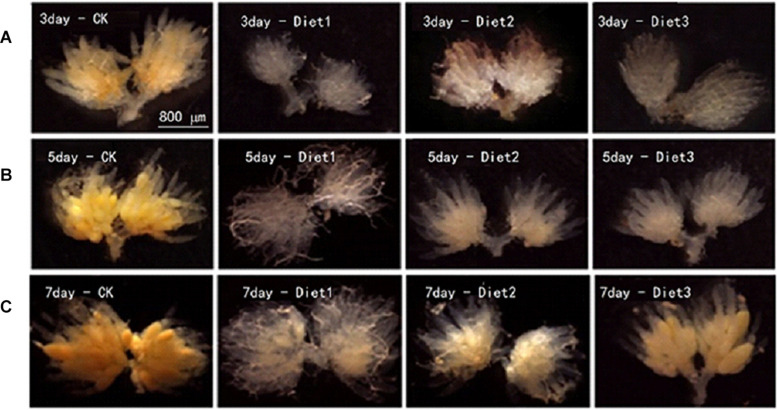
Ovaries development of *H. axyridis*. Dissecting the ovaries of female *H. axyridis* in saline, after the wings were cut off, the back of the ladybird was gently made open. Remove non-reproductive tissues and organs such as fat body and digestive tract and intact ovaries could be seen at the end of the abdomen. **(A)** The ovarian anatomy of *H. axyridis* on the 3rd day after feeding. **(B)** The ovarian anatomy of *H. axyridis* on the 5th day after feeding. **(C)** The ovarian anatomy of the *H. axyridis* after 7th day after feeding.

Our results showed that the development of *H. axyridis*’ ovaries that fed on aphids was obviously better than that of the artificialy-fed groups. The ovarian development of Diet 2 and Diet 3 insects was significantly better than that of Diet 1 insects (Figure 6).

### Relative Expression of *VgR* and *Vgs* in *H. axyridis* Adults

**FIGURE 7:**
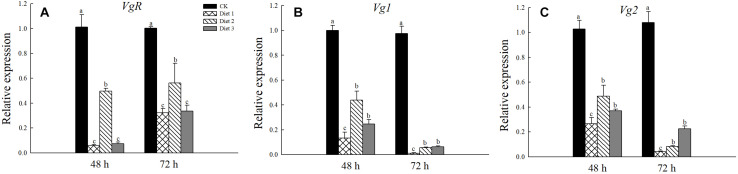
Relative expression levels of *VgR*, *Vg1*, and *Vg2* after fed for 48 and 72 h. **(A)** Relative expression of *VgR* gene. **(B)** Relative expression of *Vg1*gene. **(C)** Relative expression of *Vg2*gene. Line bars represent standard error of the mean. Bars with different letters are significantly different (Tukey–Kramer tests, *P* < 0.05).

We tested the relative expression levels of the *VgR*, *Vg1*, and *Vg2* genes in *H. axyridis* adult that fed on different artificial diets for 24 and 48 h. The *VgR* gene expression in Diet 2 individuals was significantly higher than it was in Diet 1 individuals (*P* < 0.05; Figure 7A). Additionally, there was no significant difference between the Diet 1 and Diet 3 groups. Regarding the relative expression of the *Vg1* and *Vg2* genes, the gene expression of Diet 2 and Diet 3 was significantly higher than that of Diet 1 insects. The gene expressions of all artificial feed groups were significantly lower than those of the CK group (*P* < 0.05; Figures 7B,C).

## Discussion

The results of this study showed that the trehalose, glucose, and glycogen contents in the hemolymph and tissues of larvae that fed on artificial Diet 2 and Diet 3 increased, indicating that the addition of glucose and trehalose has a certain impact on the life indicators of *H. axyridis*. Glycogen plays a regulatory role in insect development, affecting larval development and the ability to pupate (Yee et al., 2012; Puggioli et al., 2013). There are differences in the developmental time and viability of pupa and adult insects fed different artificial feedstuffs (Silva et al., 2009), this was confirmed by our study. In this study, the weight of larvae fed on Diet 2 and Diet 3 was significantly higher than that of those that fed on Diet 1. Additionally, the developmental period of larvae fed on Diet 2 and Diet 3 was extended significantly. Adding an appropriate amount of energy substances such as glucose or trehalose can help insects grow and develop. It has been reported that the health indicators of larvae affect the fecundity of adults (Leuck and Perkins, 1972).

It has been suggested that the growth and reproduction of insect populations are important indicators for analyzing and understanding insect artificial diet (Bellows et al., 1992). In this study, although the *H. axyridis* on Diet 1 reproduced and laid eggs, their fecundity was relatively lower than that of those fed on Diet 2 and Diet 3. This could be a result of the amount of nutrients and energy in their diet that were not enough for them to grow and reproduce. After adding glucose or trehalose to the pork liver diet, not only the pre-oviposition period shortened, but also the fecundity and fertility of the eggs increased. When newly hatched larvae have sufficient food resources, their mortality rate will decrease as they develop faster, which reduces damage during development (Lucas, 2005; Lebreton et al., 2015). The apparent benefits on the reproductive ability of insects that fed on artificial feedstuffs containing glucose or trehalose that were observed in the experiments described in this paper, are inconsistent with the results of a previous study, which reported that feeding *Cydia pomonella* with sugars (Siekmann et al., 2001) can extend their lives but cannot increase their or their eggs’ fertility (Savary et al., 2019). However, the results of increased fertility obtained in this study are consistent with earlier reports of increased fertility caused by feeding sugar (Fadamiro and Heimpel, 2001). In parasitic bees, the benefits of glucose on health-related characteristics such as longevity and fertility are well-documented (Desouhant et al., 2010).

Trehalose has been reported as the dominant sugar in the insect hemolymph and other tissues (Van Handel, 1969) as it provides energy for targeted activities (Lu et al., 2019). In our study, we detected that the ATP content in the Diet 2 and Diet 3 groups was significantly higher than that in the Diet 1 group. In addition, studies have reported that developing insect embryos use glycogen to generate ATP energy and produce biomolecules required for cell proliferation and differentiation to maintain the embryonic development (Tennessen et al., 2011). Further metabolic analysis has shown that glycogen stored in oocytes is consumed during embryogenesis (Matsuda et al., 2015). In this study, we found that the glycogen content of Diet 2 and Diet 3 adults was significantly higher than that of Diet 1 adults. This is relatively consistent with the results of the larval glycogen content. At the same time, the overall glucose content increased. The glucose increase could have been caused by the conversion of glycogen to glucose (Fraga et al., 2013). Prolonged starvation reduces trehalose and glucose levels (Schilman and Roces, 2008; Laparie et al., 2012). The ovary is a reproductive organ that plays a vital role in population reproduction (Koch, 2003). There is high correlation between trehalose levels and spawning. Decreasing trehalose levels will delay the production of oocysts (Huang and Lee, 2011). The results of the ovarian development map showed that the ovarian development of the Diet 1 group was significantly more stunted than that of the Diet 2 and Diet 3 groups. The development of the ovary can be based on the presence or absence of egg chambers, transparent ovaries or yolk deposition, etc., to determine whether the development is good at the normal development stage (Chen et al., 2015). This suggests that trehalose may be an important energy fuel during spawning.

The relative gene expression of *VgR*, *Vg1*, and *Vg2* of insects that fed on Diet 2 and Diet 3 was significantly higher than that of those that fed on Diet 1. Oviposition is one of the most energy-demanding activities of adult female insects. During the development of oocytes, Vg is synthesized in the fat body, secreted into hemolymph, and enters developing oocytes through endocytosis mediated by the VgR (Tufail and Takeda, 2008). Therefore, the inadequate trehalose intake by the egg cells, which could inhibit the expression of *Vg*, may be the main reason for the delayed oviposition of *H. axyridis*. Research on *Drosophila* has suggested that the higher expression rate of *Vg* (vitellogenin precursor) leads to an increase in egg maturation and fertility (Drapeau et al., 2006). Up to now, *Vg* has been widely studied in insects, with its direct physiological function being to provide nutrition for developing embryos (Tufail and Takeda, 2008). In our study, the addition of glucose or trehalose increased the *Vg* expression. Researchers have discovered that *Vg* is involved in the maturation and development of oocytes and is, therefore, a key factor in insect reproduction. Additionally, *Vg* has been studied in many insects, including Lepidoptera, Diptera, Hymenoptera, and Hemiptera (Raviv et al., 2006).

It is well-known that the activity of any insect is closely associated with its energy metabolism (Wong et al., 2016); additionally, the metabolism of fatty and amino acids affects the release of insect hormones (Van der Horst, 2003). Owing to the limited ability of the ovaries to synthesize lipids, the formation and mobilization of lipid reserves in the fat body is also critical for egg maturation. It has been reported that glucose is not only a major energy source, but also promotes the synthesis of most molecules such as amino acids, nucleotides, and fatty acids (Saltiel and Kahn, 2001). In our study, the fatty acid content of adult *H. axyridis* increased after adding glucose or trehalose to their diets (Figure 5B), which could also explain their improved ovarian development and fertility. During the development of insect oocytes, the developing oocytes accumulate a large amount of energy reserves from the hemolymph, such as lipids and proteins; essentially, reserves in the fat body that are essential for the development and reproduction of adults are mobilized (Tufail and Takeda, 2008). Excess glucose obtained from the diet is stored as branched-chain polysaccharide glycogen or as triglycerides in the body to satisfy future energy needs (Saltiel and Kahn, 2001; Chng et al., 2017). In this study, the glycerol content of the insects increased with the consumption of artificial feedstuff containing glucose or trehalose, indicating that the proper adjustment of the stored energy’s anabolism and catabolism is critical to preserve the insect’s metabolic balance throughout its life cycle (Mattila and Hietakangas, 2017). Moreover, studies have shown that insects with insufficient energy supply mobilize glycogen and triglycerides stored in their fat bodies (Bede et al., 2007; Konuma et al., 2012; Park et al., 2013). The lipid reserves of hungry females gradually decrease, while the lipid reserves of hungry males remain stable, indicating that nutrient reserves (lipids and glycogen) may be used when females spawn (Fadamiro et al., 2005). The fat content and fatty acid composition of insects depend on the composition of their feedstuffs and on the feeding conditions (Van Broekhoven et al., 2015), it follows that larvae fed low nutritional quality foods may use their fat reserves as energy, thereby reducing their fat content (St-Hilaire et al., 2007).

Therefore, the selection of a suitable artificial diet as larval food is the basis for the breeding success of *H. axyridis*. Research has indicated that there is an energetic cost to the act of reproduction (Wu et al., 2008) and several studies have shown that sugar has a positive effect on insect survival or fertility (Fadamiro and Heimpel, 2001). In addition, glucose increases mating events (Lebreton et al., 2015), which may contribute to the increase in spawning. Moreover, studies have shown that glucose can promote gastrointestinal motility, intestinal transport, and excretion (Chng et al., 2017), although these topics require further study.

## Conclusion

Our results demonstrated that glucose and trehalose contributed to the growth, development and reproduction of *H. axyridis*, which may be due to their roles in energy. The reproduction of *H. axyridis* was elevated with addition of glucose and trehalose in our study. Adult reproduction depends on energy consumption and accumulation in the early stages of insect development, and the more is energy, the strong is reproduction. Therefore, the selection of suitable artificial diet as larval food is the basis for the success of *H. axyridis* breeding.

## Data Availability Statement

All datasets generated for this study are included in the article/supplementary material.

## Author Contributions

SGW and SW conceived the design and manuscript structure design. YL, YKL, YTL, SSW, and MZ contributed to the current articles collection and trehalose metabolism genes’ analysis. YL, SW, and SGW wrote the manuscript. All authors contributed to the article and approved the submitted version.

## Conflict of Interest

The authors declare that the research was conducted in the absence of any commercial or financial relationships that could be construed as a potential conflict of interest.
